# 21-Gene Recurrence Score Assay and Outcomes of Adjuvant Radiotherapy in Elderly Women With Early-Stage Breast Cancer After Breast-Conserving Surgery

**DOI:** 10.3389/fonc.2019.00001

**Published:** 2019-01-29

**Authors:** San-Gang Wu, Wen-Wen Zhang, Jun Wang, Yong Dong, Jia-Yuan Sun, Yong-Xiong Chen, Zhen-Yu He

**Affiliations:** ^1^Department of Radiation Oncology, Cancer Hospital, The First Affiliated Hospital of Xiamen University, Teaching Hospital of Fujian Medical University, Xiamen, China; ^2^Department of Radiation Oncology, Sun Yat-sen University Cancer Center, State Key Laboratory of Oncology in South China, Collaborative Innovation Center of Cancer Medicine, Guangzhou, China; ^3^Department of Oncology, Dongguan Third People's Hospital, Affiliated Dongguan Shilong People's Hospital of Southern Medical University, Dongguan, China; ^4^Eye Institute of Xiamen University, Fujian Provincial Key Laboratory of Ophthalmology and Visual Science, Medical College, Xiamen University, Xiamen, China

**Keywords:** breast cancer, elderly, 21-gene recurrence score, radiotherapy, breast-conserving surgery

## Abstract

**Introduction:** To assess the role of the 21-gene recurrence score (RS) assay on decision-making of postoperative radiotherapy (RT) following breast-conserving surgery (BCS) in elderly women with early-stage breast cancer.

**Methods:** The 21-gene RS for elderly (≥65 years) women with stage T1–2N0M0 estrogen receptor-positive breast cancer who underwent BCS from 2004 to 2015 was obtained from the Surveillance, Epidemiology, and End Results program. We estimated the association of 21-gene RS and adjuvant RT related to breast cancer-specific survival (BCSS) using propensity score matching (PSM).

**Results:** We identified 18,456 patients, of which 15,326 (83.0%) received postoperative RT. Of identified patients, 58.9, 34.0, and 7.1% of patients had a low-, intermediate-, and high-risk RS, respectively. Receipt of postoperative RT was not related to the year of diagnosis according to the 21-gene RS groups. Multivariate analysis suggested that receipt of postoperative RT was an independent predictor of better BCSS before (hazard ratio [HR] 0.587, 95% confidence interval [CI] 0.426–0.809, *P* = 0.001) and after (HR 0.613, 95%CI 0.390–0.963, *P* = 0.034) PSM. However, subgroups analyses indicated that receipt of postoperative RT was related to better BCSS in women with intermediate-risk RS before (HR 0.467, 95%CI 0.283–0.772, *P* = 0.003) and after (HR 0.389, 95%CI 0.179–0.846, *P* = 0.017) PSM, but not in women with low- and high-risk RS groups before and after PSM.

**Conclusions:** Although causation cannot be implied, adjuvant RT in elderly women was associated with a greater effect size in patients with an intermediate-risk RS.

## Background

Several prospective studies have shown that radiotherapy (RT) following breast-conserving surgery (BCS) can improve locoregional control in elderly women with low-risk breast cancer, but this regimen has no effect on distant recurrence or overall survival (OS) (1–4). Nevertheless, more than two-thirds of elderly patients undergo RT after BCS (5, 6), which suggests that the results of prospective studies have not decreased use of postoperative RT significantly in this population. Comorbidities are common among elderly patients, but life expectancy could be increased with better management of such comorbidities (7–10). Therefore, valuable decision-making tools to identify elderly patients with breast cancer who may benefit from postoperative RT are required. However, authoritative decision-making tools in this population are lacking.

The 21-gene recurrence score (RS) assay (Oncotype DX; Genomic Health, Redwood City, CA, USA) is used to assess the breast-cancer genes related to proliferation and invasion. The 21-gene RS assay can also provide information on the risk of distant recurrence as well as the benefit of adjuvant chemotherapy (11–13). In addition, several recent studies have shown that the 21-gene RS is correlated with the risk of locoregional recurrence (LRR). That is, a high RS is associated with a high risk of LRR in patients with node-negative or node-positive estrogen receptor (ER)-positive breast cancer (14, 15).

Studies focusing on whether the 21-gene RS assay can be used to identify a subgroup of elderly women with breast cancer for whom RT following BCS is not indicated are lacking. We hypothesized that postoperative RT could provide greater survival benefit for patients with a high RS. Therefore, we investigated the potential role of the 21-gene RS assay on prediction of RT outcome in elderly women with breast cancer after BCS.

## Materials and Methods

### Database and Patients

This retrospective study included data derived from Surveillance, Epidemiology, and End Results Program (SEER) database, the SEER database contains anonymized data of cancer incidence, patient demographics, first course of treatment, and survival outcomes of ~28% of the USA population. We linked to the National Cancer Institute's SEER 18 Regs (Excl AK) Custom Data Malignant Breast (with Oncotype DX and Additional Treatment Fields) to include breast cancer patients with available 21-gene RS testing between 2004 and 2015 (16). The results of 21-gene RS assay were provided by Genomic Health Clinical Laboratory, and were released in 2018 in the SEER database. The permission to access this database for the present study was authorized.

We identified women with 21-gene RS data who: (i) were aged ≥65 years with invasive breast cancer; (ii) had or did not have adjuvant RT following BCS; (iii) had stage tumor size ≤ 5 cm and node-negative disease (T1–T2N0); (iv) had ER-positive disease. Patients with no positive pathology diagnosis, receipt of preoperative RT, or an unknown sequence of RT and surgery were excluded. We did not need to obtain written informed consent from our institution because SEER data are anonymized.

### Variables

We were interested in the following variables: 21-gene RS, RT, age, tumor grade, race/ethnicity, histology subtype, tumor stage, and chemotherapy. The groups of 21-gene RS were classified as having a “low-risk” (RS < 18), “intermediate-risk” (17–29), or “high-risk” (>30) (30). Breast cancer-specific survival (BCSS) was the primary endpoint of this study and was defined as the time from diagnosis to death from breast cancer.

### Statistical Analyses

The variables of patient demographic, clinicopathological, and treatment variables, were compared using the chi-squared test or Fisher's exact test upon receipt of postoperative RT. To reduce the potential confounding of retrospective studies, a 1:1 match including the variables mentioned above was conducted using propensity score matching (PSM) to create matched cohorts (17, 18). BCSS curves were plotted using the Kaplan–Meier method and then compared with the log-rank test. Multivariate Cox proportional hazards models with the backward Wald method were used to assess the independent prognostic indicators related to BCSS. The hazard ratios (HRs) and their corresponding 95% confidence intervals (CIs) were calculated. Statistical analyses were carried out using SPSS 22.0 (IBM, Armonk, NY, USA). *P* < 0.05 was considered statistically significant.

## Results

We identified 18,456 patients (median age, 69 years; range, 65–93 years). The baseline characteristics of RT and non-RT groups are listed and compared in Table 1. A total of 15,326 patients (83.0%) received postoperative RT. Patients with younger age (*P* < 0.001), non-Hispanic White (*P* < 0.001), and moderately differentiated (*P* = 0.013) were more likely to receive postoperative RT. We found that 11.8% of patients had chemotherapy, and patients who received postoperative RT were more likely to receive chemotherapy (*P* = 0.048). Of the identified patients, 58.9, 34.0, and 7.1% of patients were classified as having a low-, intermediate-, and high-risk RS, respectively. In addition, patients with low-risk RS group were more likely to receive postoperative RT (*P* = 0.001). Moreover, patients with a higher RS were more likely to receive chemotherapy (*P* < 0.001)

**Table 1:** Patient characteristics in the entire cohort before and after propensity score matching.

**Variables**	**Before PSM**				**After PSM**			
	***n***	**Non-RT (%)**	**RT (%)**	***P***	***n***	**Non-RT**	**RT**	***P***
**AGE (YEARS)**								
65–74	15,201	2,305 (73.6)	12,896 (84.1)	< 0.001	4554	2277	2277	1
≥75	3,255	825 (26.4)	2,430 (15.9)		1590	795	795	
**RACE/ETHNICITY**								
Non-Hispanic White	14,855	2,416 (77.2)	12,439 (81.2)	< 0.001	4,816	2,408	2,408	1
Non-Hispanic Black	1,359	263 (8.4)	1,096 (7.2)		484	242	242	
Hispanic (all races)	1,188	265 (8.5)	923 (6.0)		490	245	245	
Other	1,054	186 (5.9)	868 (5.7)		354	177	177	
**GRADE**								
Well differentiated	5,089	853 (27.3)	4,236 (27.6)	0.013	1,684	842	842	1
Moderately differentiated	10,002	1,644 (52.5)	8,358 (54.5)		3,244	1,622	1,622	
Poorly/undifferentiated	2,929	546 (17.4)	2,383 (15.5)		1,068	534	534	
Unknown	436	87 (2.8)	349 (2.3)		148	74	74	
**HISTOLOGY SUBTYPE**								
Infiltrating ductal carcinoma	13,406	2,249 (71.9)	11,157 (72.8)	0.151	4,466	2,233	2,233	1
Lobular carcinoma	2,157	355 (11.3)	1,802 (11.8)		660	330	330	
Other	2,893	526 (16.8)	2,367 (15.4)		1,018	509	509	
**TUMOR STAGE**								
T1	14,544	2,457 (78.5)	12,087 (78.9)	0.647	4,838	2,419	2,419	1
T2	3,912	673 (21.5)	3,239 (21.1)		1,306	653	653	
**CHEMOTHERAPY**								
No/unknown	16,283	2,794 (89.3)	13,489 (89.3)	0.048	5,500	2,750	2,750	1
Yes	2,173	336 (10.7)	1,837 (10.7)		644	322	322	
**21-GENE RS**								
Low-risk	10,878	1,788 (57.1)	9,090 (59.3)	0.001	3,544	1,772	1,772	1
Intermediate-risk	6,266	1,072 (34.2)	5,194 (33.9)		2,094	1,047	1,047	
High-risk	1,312	270 (8.6)	1,042 (6.8)		506	253	253	

**Figure 1:**
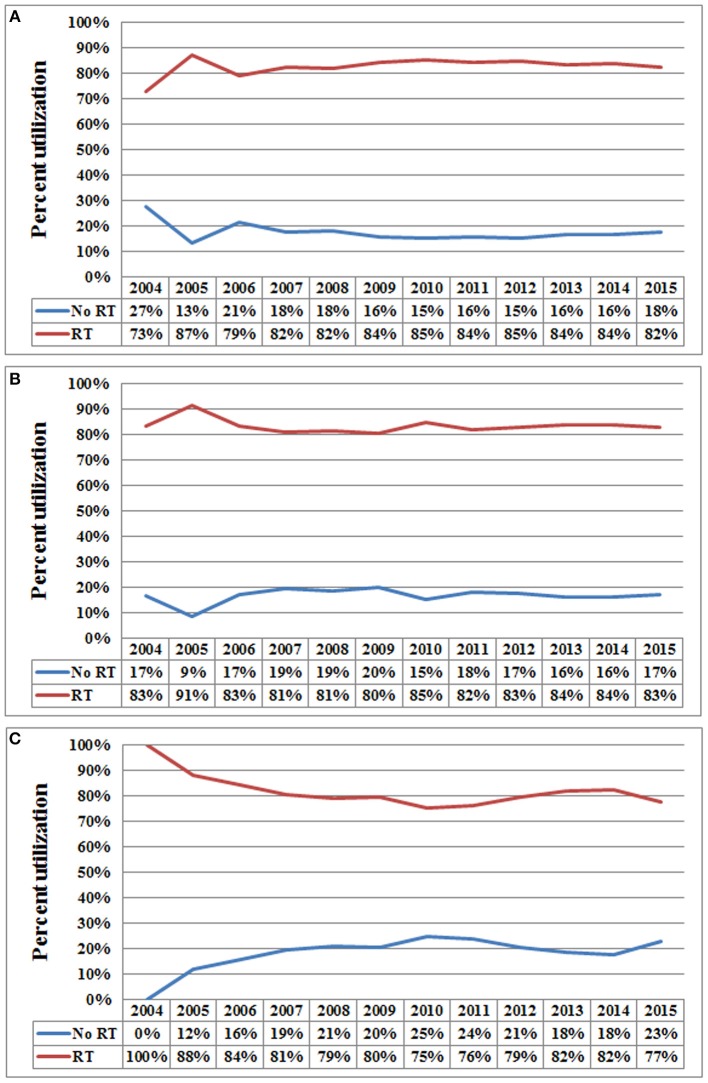
Use of postoperative radiotherapy vs. non-use of postoperative radiotherapy over time (**A**, low-risk RS group; **B**, intermediate-risk RS group; **C**, high-risk RS group).

**Figure 2:**
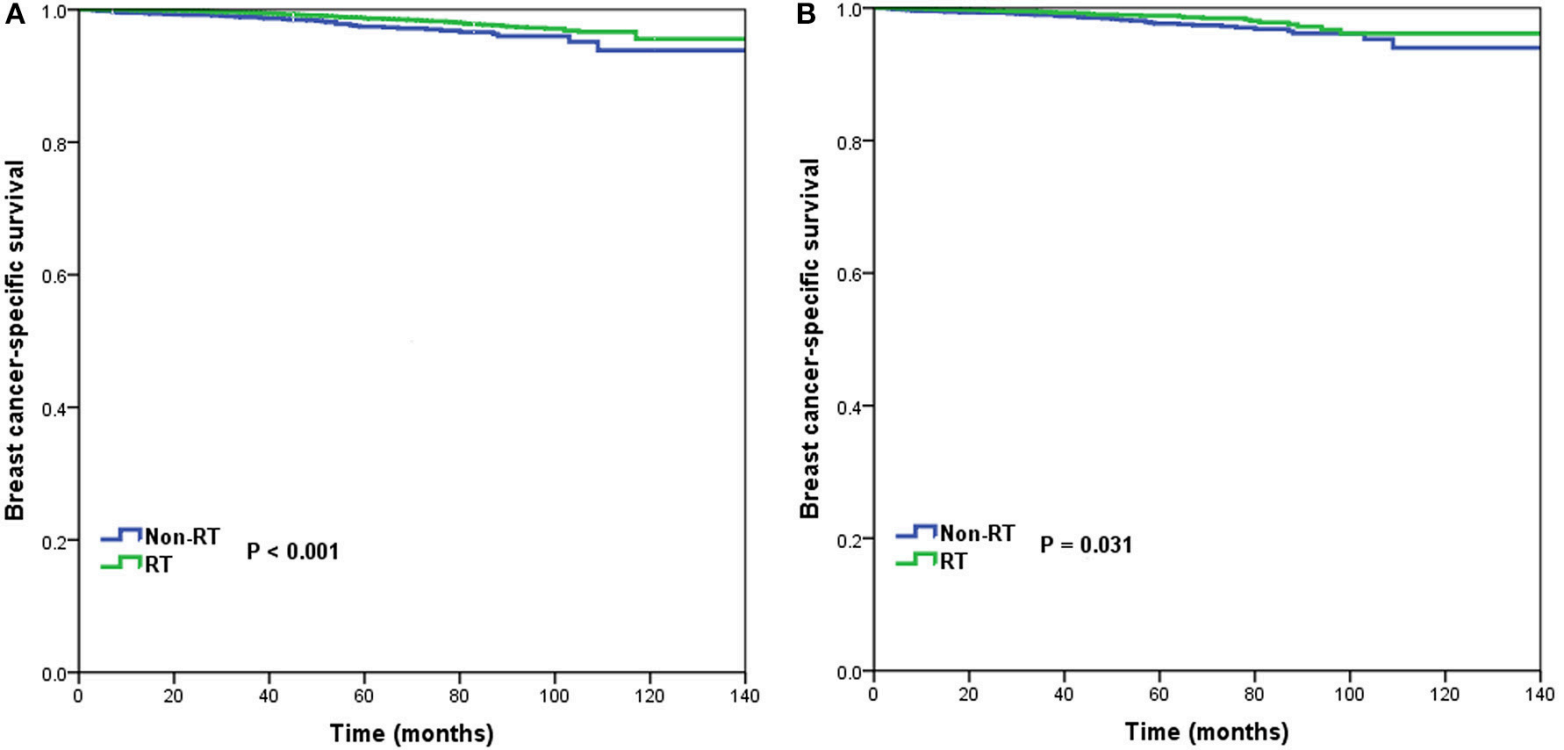
Breast cancer-specific survival in patients who had or did not have postoperative radiotherapy in the entire cohort before **(A)** and after **(B)** propensity score matching.

In the entire cohort, receipt of postoperative RT was not associated with the year of diagnosis (*P* = 0.275). In addition, in the low- (*P* = 0.302) (Figure 1A), intermediate- (*P* = 0.512) (Figure 1B), and high-risk RS groups (*P* = 0.849) (Figure 1C), receipt of postoperative RT was also not related to the year of diagnosis. Five-year BCSS was 98.5% with a median follow-up of 38 (range, 0–142) months. Postoperative RT was related to better BCSS. Five-year BCSS was 97.4 and 98.7% in non-RT and RT groups, respectively (*P* < 0.001) (Figure 2A). Multivariate analysis suggested that receipt of adjuvant RT was an independent prognostic factor related to better BCSS (HR 0.587, 95%CI 0.426–0.809, *P* = 0.001). Age, tumor grade, tumor stage, and 21-gene RS were also the independent prognostic indicators for BCSS (Table 2).

**Table 2:** Multivariate prognostic analyses before and after propensity score matching.

**Variables**	**Before PSM**			**After PSM**		
	**HR**	**95%CI**	***P***	**HR**	**95%CI**	***P***
**AGE (YEARS)**						
65–74	1			1		
≥75	1.455	1.036-2.045	0.030	1.454	0.876-2.412	0.148
**RACE/ETHNICITY**						
Non-Hispanic White	1			1		
Non-Hispanic Black	1.577	0.997–2.493	0.052	1.152	0.720–3.175	0.275
Hispanic (all races)	1.111	0.616–2.002	0.727	0.929	0.371–2.327	0.875
Other	0.760	0.356–1.622	0.478	0.517	0.126–2.117	0.356
Grade						
Well differentiated	1			1		
Moderately differentiated	2.389	1.402–4.071	0.001	2.281	1.106–5.119	0.046
Poorly/undifferentiated	3.060	1.716–5.459	< 0.001	2.305	0.940–5.563	0.068
Unknown	2.230	0.814–6.107	< 0.001	2.244	0.457–11.011	0.319
**HISTOLOGY SUBTYPE**						
Infiltrating ductal carcinoma	1			1		
Lobular carcinoma	0.896	0.533–1.507	0.680	1.076	0.500–2.315	0.852
Other	0.816	0.518–1.285	0.380	0.804	0.395–1.634	0.546
**TUMOR STAGE**						
T1	1			1		
T2	1.589	1.172–2.154	0.003	1.249	0.759–2.055	0.382
**CHEMOTHERAPY**						
No/unknown	1			1		
Yes	0.888	0.610–1.293	0.535	1.054	0.588–1.892	0.859
**21-GENE RS**						
Low-risk	1			1		
Intermediate-risk	1.873	1.307–2.682	0.001	2.205	1.267–3.837	0.005
High-risk	6.108	4.085–9.131	< 0.001	8.021	4.534–14.190	<0.001
**RT**						
No	1			1		
Yes	0.587	0.426–0.809	0.001	0.613	0.390–0.963	0.034

A total of 3,072 pairs of patients were matched completely. Patient characteristics after PSM are listed in Table 1. Receipt of postoperative RT was related to better BCSS. Five-year OS was 97.7 and 98.8% in non-RT and RT groups, respectively (*P* = 0.031) (Figure 2B). Receipt of postoperative RT was an independent prognostic indicator related to better BCSS (HR 0.613, 95%CI 0.390–0.963, *P* = 0.034) in multivariate analysis after PSM (Table 2).

**Figure 3:**
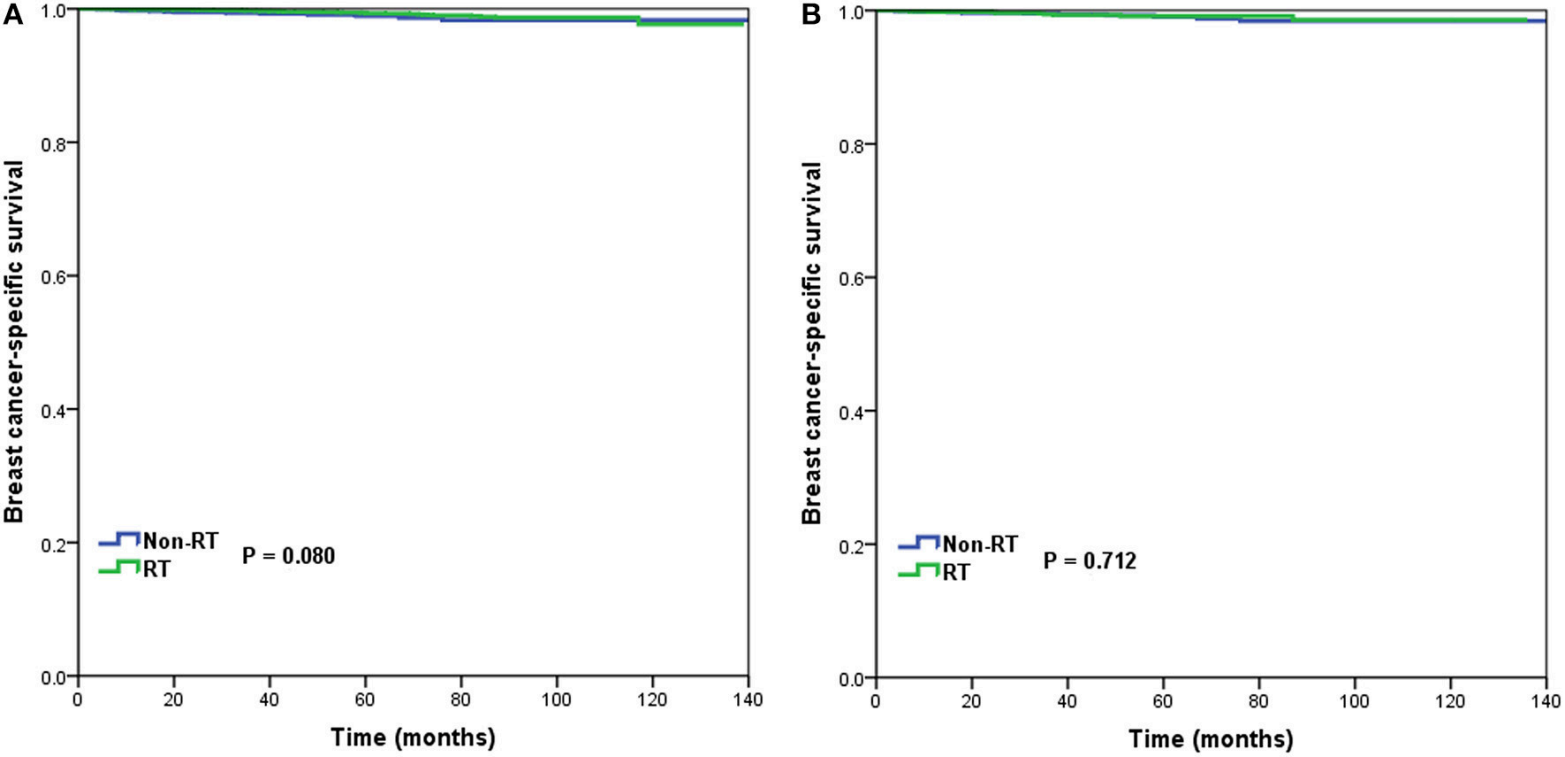
Breast cancer-specific survival in patients who had or did not have postoperative radiotherapy in the low-risk group before **(A)** and after **(B)** propensity score matching.

In 10,878 patients of the low-risk RS group, 9,090 (83.6%) patients had postoperative RT. A total of 1,772 pairs of patients were matched completely. Patient characteristics before and after PSM are listed in Supplemental Table 1. The administration of postoperative RT was not associated with better BCSS before (5-year BCSS was 98.9 and 99.3% in non-RT and RT groups, respectively, *P* = 0.080) (Figure 3A) and after (5-year BCSS was 99.1 and 99.2% in non-RT and RT groups, respectively, *P* = 0.712) (Figure 3B) PSM. Multivariate analysis also indicated that receipt of postoperative RT was not related to better BCSS before (HR 0.653, 95%CI 0.347–1.227, *P* = 0.186) and after (HR 0.836, 95%CI 0.354–1.973, *P* = 0.683) PSM (Table 3).

**Table 3:** Multivariate prognostic analyses according to RS before and after propensity score matching.

**21-gene RS groups**	**Before PSM**			**After PSM**		
	**HR**	**95%CI**	***P***	**HR**	**95%CI**	***P***
**LOW-RISK**						
Non-RT	1			1		
RT	0.653	0.347–1.227	0.186	0.836	0.354–1.973	0.683
**INTERMEDIATE-RISK**						
Non-RT	1			1		
RT	0.467	0.283-0.772	0.003	0.389	0.179-0.846	0.017
**HIGH-RISK**						
Non-RT	1			1		
RT	0.797	0.454–1.401	0.431	0.766	0.358–1.639	0.492

**Figure 4:**
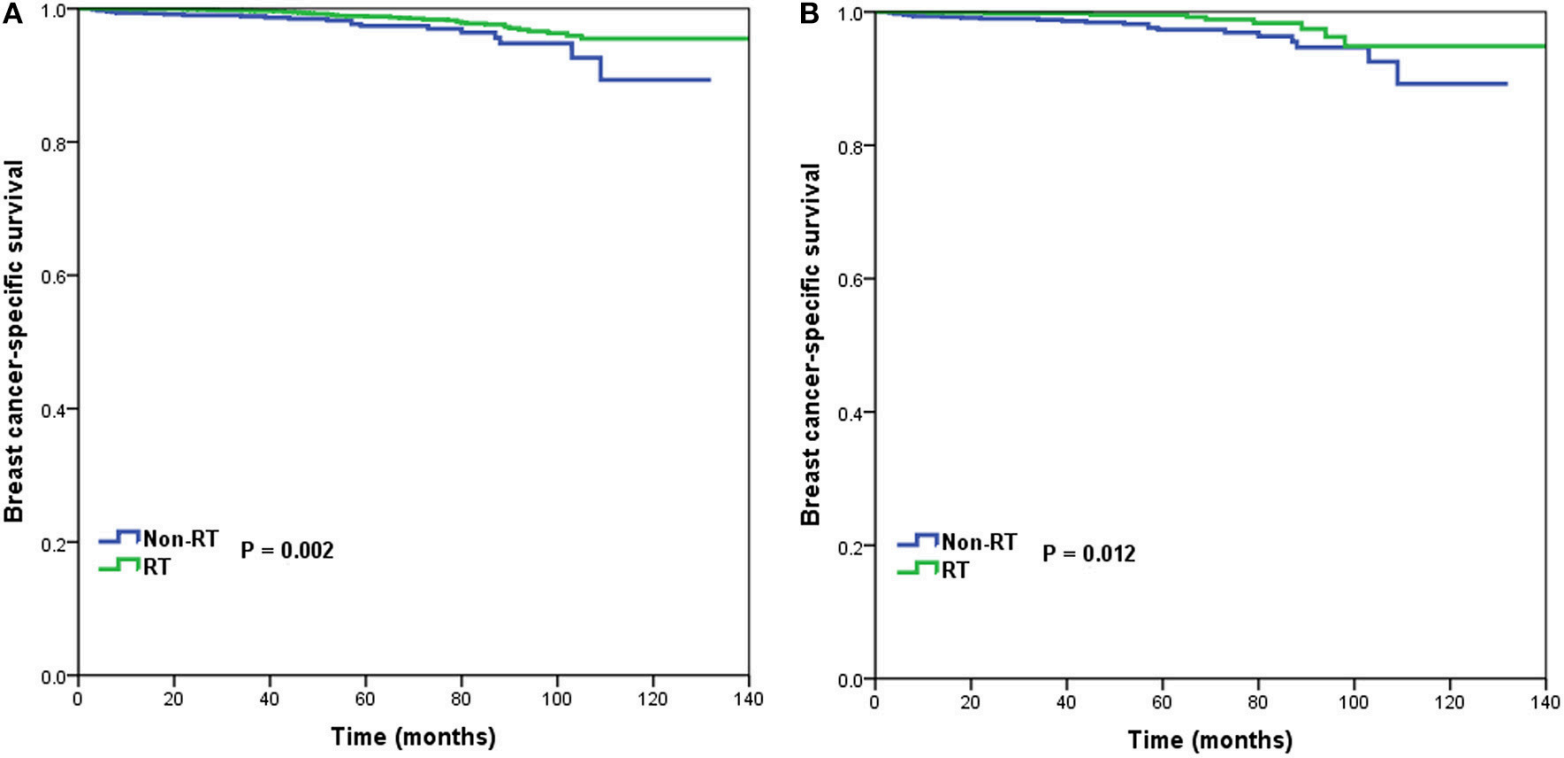
Breast cancer-specific survival in patients who had or did not have postoperative radiotherapy in the intermediate-risk group before **(A)** and after **(B)** propensity score matching.

In 6,266 patients of the intermediate-risk RS group, 6,194 (82.9%) patients received postoperative RT. A total of 1,047 pairs of patients were matched completely. Patient characteristics before and after PSM are listed in Supplemental Table 2. Before PSM, receipt of postoperative RT was correlated with better BCSS. Five-year BCSS was 97.4 and 98.8% in non-RT and RT groups, respectively (*P* = 0.002) (Figure 4A). There was an absolute BCSS benefit of 2.3% in the RT group compared with the non-RT group after PSM (99.6 vs. 97.3%, *P* = 0.012) (Figure 4B). Multivariate analysis showed that receipt of postoperative RT was independently related to better BCSS before (HR 0.467, 95%CI 0.283–0.772, *P* = 0.003) and after (HR 0.389, 95%CI 0.179–0.846, *P* = 0.017) PSM (Table 3).

**Figure 5:**
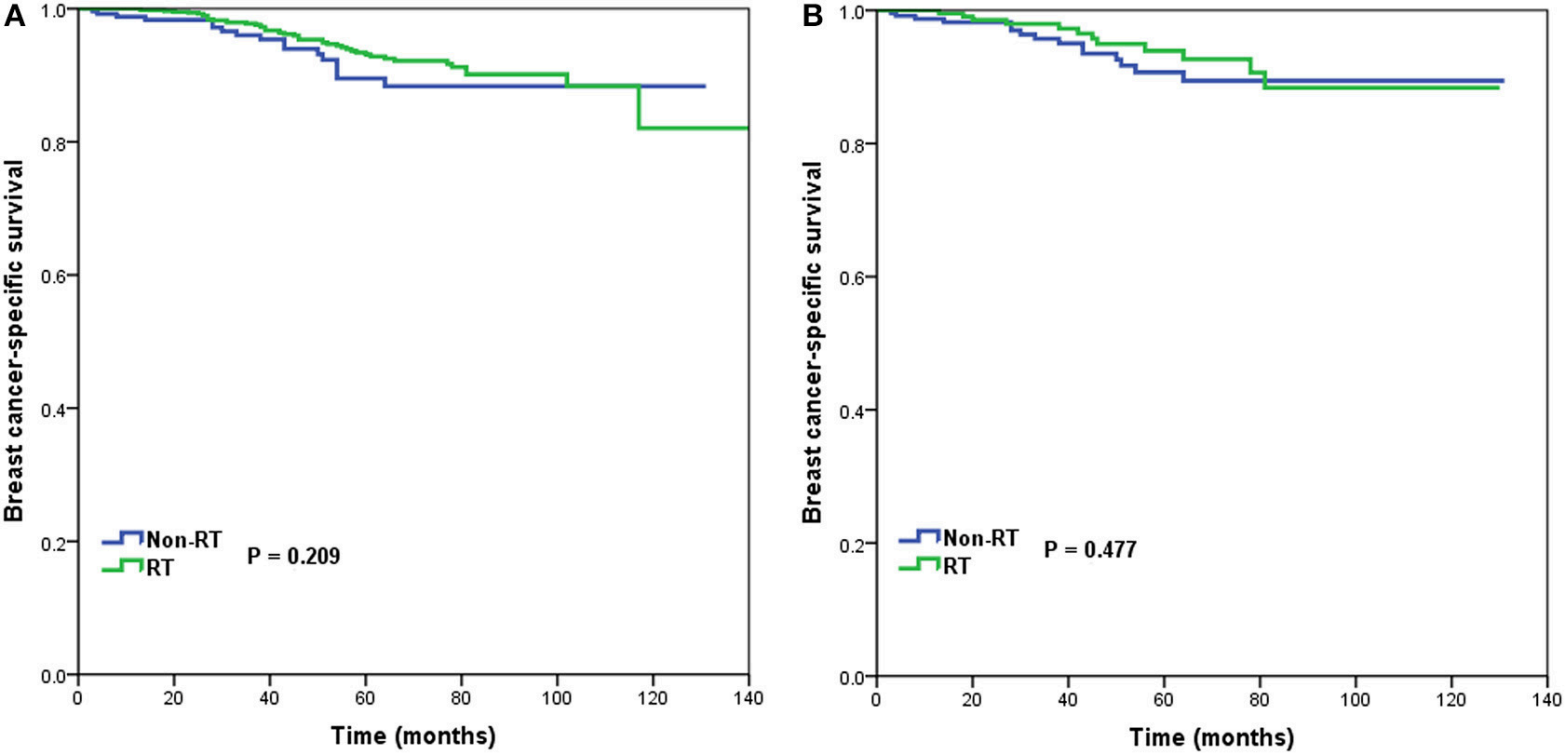
Breast cancer-specific survival in patients who had or did not have postoperative radiotherapy in the high-risk group before **(A)** and after **(B)** propensity score matching.

In 1,312 patients of the high-risk RS group, 1,042 (79.4%) patients received postoperative RT. A total of 253 pairs of patients were matched completely. Patients characteristics before and after PSM are listed in Supplemental Table 3. Before PSM, receipt of postoperative RT was not associated with better BCSS. Five-year BCSS was 89.5 and 93.1% in non-RT and RT groups, respectively (*P* = 0.209) (Figure 5A). There was also comparable BCSS between non-RT and RT groups after PSM (90.7 vs. 93.9%, *P* = 0.477) (Figure 5B). Multivariate analysis showed that receipt of postoperative RT did not independently impact BCSS before (HR 0.797, 95%CI 0.454–1.401, *P* = 0.431) and after (HR 0.766, 95%CI 0.358–1.639, *P* = 0.492) PSM (Table 3).

## Discussion

We undertook a population-based study to assess the role of the RS in predicting the benefit of RT following BCS in elderly women with breast cancer. Our results suggested that postoperative RT was related to better BCSS in intermediate-risk RS group, whereas women with low- and high-risk RS groups who received RT after BCS had no BCSS benefit compared with women who did not receive postoperative RT.

The results from Early Breast Cancer Trialists' Collaborative Group have indicated that the administration of postoperative RT after BCS was associated with improving locoregional control and OS even in the younger age groups (19, 20). In addition, several randomized trials have demonstrated that receipt of RT following BCS in elderly patients is associated with better locoregional control. However, the improvement in LRR did not translate into an advantage in distant disease-free survival and OS (1–4). The results of those studies have a minimal impact on the use of postoperative RT among elderly women with a low risk of breast cancer after BCS. There were 67.5–89% of patients still receipt of adjuvant RT after the Cancer and Leukemia Group B (CALGB) 9343 publication (5, 6), which was similar to our study. In addition, we found that receipt of postoperative RT improved BCSS compared with patients who did not have postoperative RT, data that are similar to results from two National Cancer Database (NCDB) studies (21, 22). Therefore, elderly patients for whom postoperative RT can be omitted safely should be investigated further.

The results of 21-gene RS testing could predict the risk of distant recurrence. There were several studies also indicated that the 21-gene RS was related to the risk of LRR in patients with node-negative or node-positive disease (14, 15, 23). The 10-year LRR was 3.3–12.5, 5.1–27.7, and 12.0–26.5% in patients with low-, intermediate-, and high-risk RS groups, respectively (14, 15, 23). Therefore, it is hypothesized that adjuvant RT after BCS may provide the greatest survival benefit in patients with a higher RS.

No studies have assessed the role of the 21-gene RS assay in predicting the benefit of RT following BCS in elderly women with breast cancer. A recent study by Goodman et al. included a large cohort of T1–2N1 patients who underwent mastectomy from NCDB and SEER databases. Their results suggested that longer OS associated with postmastectomy RT was limited to women with a low-risk RS, but not for women with an intermediate- or high-risk RS. Also, the 5-year OS benefit in the low-risk RS group who received postmastectomy RT was ~2–3% (24). However, they did not analyze the results of BCSS. Since there were tons of confounding factors associated with OS. The analysis of BCSS would be much more robust than OS in the SEER database. A recent study by Jayasekera et al. included 1,778 patients aged 40–74 years from seven trials, they showed that the omission of RT in low-risk RS group who treated with hormonal therapy may lead to small absolute differences in LRR (5-year LRR 6.3 and 1.4% in non-RT and RT groups, respectively), but does not appear to increase the risk of distant recurrence or death (25). In our study, we found that receipt of postoperative RT was related to better BCSS in patients with intermediate-risk RS, the BCSS advantage among women with intermediate-risk RS who received postoperative RT was 2.3% at 5 years. However, receipt of postoperative RT was not related to better BCSS in patients with low- and high-risk RS before and after PSM. Therefore, in the modern clinical management of breast cancer, the multigene assays maybe available to determine the subgroups who can safely omit RT after BCS.

There were several potentially reasonable reasons to explain our findings. First, the risk of LRR and distant recurrence was extremely low in low-risk RS group (11–15, 23), even in patients who did not receive adjuvant RT (25). However, patients in the high-risk RS cohort who are at highest risk for subclinical micrometastatic disease may not be able to benefit from adjuvant RT because of the competitive risk of distant recurrence (24). Therefore, postoperative RT may sterilization of potential residual disease and finally has the greatest survival benefit to patients with intermediate-risk RS. The Danish Breast Cancer Cooperative Group 82 b&c trials also showed that patients with intermediate-risk of LRR were associated with the significantly benefit in LRR, disease specific survival, and OS after receiving postmastectomy RT. However, the benefit in LRR did not translate into disease specific survival and OS benefit in the low- and high-risk groups (26). Second, this effect may be related to differences in radiosensitivity between molecular subtypes; patients with intermediate-risk of LRR may have better outcomes due to higher radiosensitivity (27). In addition, the translation from LRR benefit to BCSS benefit appears to be heterogeneous and varies between subpopulations on the basis of distant-recurrence risk and/or intrinsic radiosensitivity. Finally, it is hypothesized that adjuvant RT may provide the greatest benefit in women with a high-risk RS. The opposite result of our study maybe due to the higher percentage of chemotherapy receipt in high-risk RS group, and thus the effect of adjuvant RT was diluted.

Validated tools to help clinicians determine which elderly women will benefit from adjuvant RT are lacking (28, 29). The RS assay can quantify the risk of distant metastasis and predict the benefit from adjuvant chemotherapy (31, 32). However, in our study, receipt of postoperative RT was not related to the year of diagnosis according to the RS assay. Nevertheless, our results suggest that the RS may be useful for identifying elderly patients who may benefit from RT after BCS.

Our study had several limitations. First, although we used PSM to reduce potential confounding effects, there are potential inherent biases in retrospective studies. Second, patients who did not receive RT may have had more complications that affect the receipt of radiotherapy. However, the SEER database does not contain information on patients' complications. Third, the details of the target volume, dose, and technology of RT were lacking, and the patterns of LRR or distant metastasis are not in the SEER database. Moreover, it has been shown that there are several inaccuracies in the SEER database, with a high prevalence of under-reporting of receipt of radiotherapy and chemotherapy (33, 34).

## Conclusion

In conclusion, although causation cannot be implied, adjuvant RT in elderly women was associated with a greater effect size in patients with an intermediate-risk RS. Our results caution against omission of RT after BCS for elderly women with a low risk of LRR. More prospective studies are required to confirm our findings.

## Ethics Statement

The study was exempt from the approval processes of the Institutional Review Boards because the SEER database patient information is de-identified.

## Author Contributions

S-GW and W-WZ are lead authors who participated in data collection, manuscript drafting, table and figure creation, and manuscript revision. JW, YD, and J-YS are senior authors who aided in drafting the manuscript and manuscript revision. Y-XC and Z-YH is the corresponding author who initially developed the concept and drafted and revised the manuscript. All authors read and approved the final manuscript.

### Conflict of Interest Statement

The authors declare that the research was conducted in the absence of any commercial or financial relationships that could be construed as a potential conflict of interest.
