# Supplementary material for: 21-Gene Recurrence Score Assay and Outcomes of Adjuvant Radiotherapy in Elderly Women With Early-Stage Breast Cancer After Breast-Conserving Surgery
Source: Front Oncol. 2019 Jan 29;9:1. doi: 10.3389/fonc.2019.00001 (PMC6361832; doi:10.3389/fonc.2019.00001)
Supplement: Supplementary file 1 [file Data_Sheet_1.doc]

**Supplemental tables**

**Supplemental Table 1.** Patient characteristics in the low-risk recurrence score group

before and after propensity score matching.

| Variables | Before PSM | | | | After PSM | | | |
| --- | --- | --- | --- | --- | --- | --- | --- | --- |
|  | n | Non-RT (%) | RT (%) | P | n | Non-RT | RT | P |
| Age (years) |  |  |  |  |  |  |  |  |
| 65–74 | 8953 | 1278 (71.5) | 7675 (84.4) | <0.001 | 2536 | 1268 | 1268 | 1 |
| ≥75 | 1925 | 510 (28.5) | 1415 (15.6) |  | 1008 | 504 | 504 |  |
| Race/ethnicity | |  |  |  |  |  |  |  |
| Non-Hispanic White | 8797 | 1385 (77.5) | 7412 (81.5) | <0.001 | 2770 | 1385 | 1385 | 1 |
| Non-Hispanic Black | 767 | 148 (8.3) | 619 (6.8) |  | 284 | 142 | 142 |  |
| Hispanic (all races) | 676 | 145 (8.1) | 531 (5.8) |  | 276 | 138 | 138 |  |
| Other | 638 | 110 (6.2) | 528 (5.8) |  | 214 | 107 | 107 |  |
| Grade |  |  |  |  |  |  |  |  |
| Well differentiated | 3586 | 601 (33.6) | 2985 (32.8) | 0.151 | 1196 | 598 | 598 | 1 |
| Moderately differentiated | 6089 | 970 (54.3) | 5119 (56.3) |  | 1934 | 967 | 967 |  |
| Poorly/undifferentiated | 936 | 162 (9.1) | 774 (8.5) |  | 322 | 161 | 161 |  |
| Unknown | 267 | 55 (3.1) | 212 (23.3) |  | 92 | 45 | 45 |  |
| Histology subtype |  |  |  |  |  |  |  |  |
| Infiltrating ductal carcinoma | 7754 | 1251 (67.0) | 6503 (71.5) | 0.003 | 2492 | 1246 | 1246 | 1 |
| Lobular carcinoma | 1192 | 173 (9.7) | 1019 (11.2) |  | 342 | 171 | 171 |  |
| Other | 1932 | 364 (20.4) | 1568 (17.2) |  | 710 | 355 | 355 |  |
| Tumor stage |  |  |  |  |  |  |  |  |
| T1 | 8718 | 1442 (80.6) | 7276 (80.0) | 0.558 | 2862 | 1431 | 1431 | 1 |
| T2 | 2160 | 346 (19.4) | 1814 (20.0) |  | 682 | 341 | 341 |  |
| Chemotherapy |  |  |  |  |  |  |  |  |
| No/unknown | 10696 | 1765 (98.7) | 8931 (98.3) | 0.163 | 3506 | 1754 | 1754 | 1 |
| Yes | 182 | 23 (1.3) | 159 (1.7) |  | 36 | 18 | 18 |  |

**Supplemental Table 2.** Patient characteristics in the intermediate-risk recurrence score group before and after propensity score matching.

| Variables | Before PSM | | | | After PSM | | | |
| --- | --- | --- | --- | --- | --- | --- | --- | --- |
|  | n | Non-RT (%) | RT (%) | P | n | Non-RT | RT | P |
| Age (years) |  |  |  |  |  |  |  |  |
| 65–74 | 5169 | 815 (76.0) | 4354 (83.8) | <0.001 | 1616 | 808 | 808 | 1 |
| ≥75 | 1097 | 257 (24.0) | 840 (16.2) |  | 478 | 239 | 239 |  |
| Race/ethnicity |  |  |  |  |  |  |  |  |
| Non-Hispanic White | 5023 | 820 (76.5) | 4203 (80.9) | 0.001 | 1634 | 817 | 817 | 1 |
| Non-Hispanic Black | 472 | 86 (8.0) | 386 (7.4) |  | 154 | 77 | 77 |  |
| Hispanic (all races) | 429 | 103 (9.6) | 326 (6.3) |  | 186 | 93 | 93 |  |
| Other | 342 | 63 (5.9) | 279 (5.4) |  | 120 | 60 | 60 |  |
| Grade |  |  |  |  |  |  |  |  |
| Well differentiated | 1443 | 246 (22.9) | 1197 (23.0) | 0.439 | 478 | 239 | 239 | 1 |
| Moderately differentiated | 3435 | 569 (53.1) | 2866 (55.2) |  | 1122 | 561 | 561 |  |
| Poorly/undifferentiated | 1242 | 229 (21.4) | 1013 (19.5) |  | 444 | 222 | 222 |  |
| Unknown | 146 | 28 (2.6) | 118 (2.3) |  | 50 | 25 | 25 |  |
| Histology subtype |  |  |  |  |  |  |  |  |
| Infiltrating ductal carcinoma | 4507 | 764 (71.3) | 3743 (72.1) | 0.414 | 1522 | 761 | 761 | 1 |
| Lobular carcinoma | 916 | 170 (15.9) | 746 (14.4) |  | 306 | 153 | 153 |  |
| Other | 843 | 138 (12.9) | 705 (13.6) |  | 266 | 133 | 133 |  |
| Tumor stage |  |  |  |  |  |  |  |  |
| T1 | 4930 | 842 (78.5) | 4088 (78.7) | 0.906 | 1644 | 822 | 822 | 1 |
| T2 | 1336 | 230 (21.5) | 1106 (21.3) |  | 450 | 225 | 225 |  |
| Chemotherapy |  |  |  |  |  |  |  |  |
| No/unknown | 5137 | 902 (84.1) | 4235 (81.5) | 0.043 | 1768 | 884 | 884 | 1 |
| Yes | 1129 | 170 (15.9) | 959 (18.5) |  | 326 | 163 | 163 |  |

**Supplemental Table 3.** Patient characteristics in the high-risk recurrence score group before and after propensity score matching.

| Variable | Before PSM | | | | After PSM | | | |
| --- | --- | --- | --- | --- | --- | --- | --- | --- |
|  | n | Non-RT (%) | RT (%) | P | n | Non-RT | RT | P |
| Age (years) |  |  |  |  |  |  |  |  |
| 65–74 | 1079 | 212 (78.5) | 867 (83.2) | 0.073 | 402 | 201 | 201 | 1 |
| ≥75 | 233 | 58 (21.5) | 175 (16.8) |  | 104 | 52 | 52 |  |
| Race/ethnicity |  |  |  |  |  |  |  |  |
| Non-Hispanic White | 1035 | 211 (78.1) | 824 (79.1) | 0.710 | 412 | 206 | 206 | 1 |
| Non-Hispanic Black | 120 | 29 (10.7) | 91 (8.7) |  | 46 | 23 | 23 |  |
| Hispanic (all races) | 83 | 17 (6.3) | 66 (6.3) |  | 28 | 14 | 14 |  |
| Other | 74 | 13 (4.8) | 61 (5.9) |  | 20 | 10 | 10 |  |
| Grade |  |  |  |  |  |  |  |  |
| Well differentiated | 60 | 6 (2.2) | 54 (5.2) | 0.186 | 10 | 5 | 5 | 1 |
| Moderately differentiated | 478 | 105 (38.9) | 373 (35.8) |  | 188 | 94 | 94 |  |
| Poorly/undifferentiated | 751 | 155 (57.4) | 596 (57.2) |  | 302 | 151 | 151 |  |
| Unknown | 23 | 4 (1.5) | 19 (1.8) |  | 6 | 3 | 3 |  |
| Histology subtype |  |  |  |  |  |  |  |  |
| Infiltrating ductal carcinoma | 1145 | 234 (86.7) | 911 (87.4) | 0.788 | 452 | 226 | 226 | 1 |
| Lobular carcinoma | 49 | 12 (4.4) | 37 (3.6) |  | 12 | 6 | 6 |  |
| Other | 118 | 24 (8.9) | 94 (9.0) |  | 42 | 21 | 21 |  |
| Tumor stage |  |  |  |  |  |  |  |  |
| T1 | 896 | 173 (64.1) | 723 (69.4) | 0.915 | 332 | 166 | 166 | 1 |
| T2 | 416 | 97 (35.9) | 319 (30.6) |  | 174 | 87 | 87 |  |
| Chemotherapy |  |  |  |  |  |  |  |  |
| No/unknown | 450 | 127 (47.0) | 323 (31.0) | <0.001 | 224 | 112 | 112 | 1 |
| Yes | 862 | 143 (53.0) | 719 (69.0) |  | 282 | 141 | 141 |  |
